# Planning and developing a web-based intervention for active surveillance in prostate cancer: an integrated self-care programme for managing psychological distress

**DOI:** 10.1186/s40814-022-01124-x

**Published:** 2022-08-09

**Authors:** Stephanie Hughes, Angelos P. Kassianos, Hazel A. Everitt, Beth Stuart, Rebecca Band

**Affiliations:** 1 Primary Care Population Sciences and Medical Education, University of Southampton, Southampton, UK; 2 Department of Nursing, Cyprus University of Technology, Limassol, Cyprus; 3 Department of Applied Health Research, University College London, London, UK; 4 Health Sciences, University of Southampton, Southampton, UK

**Keywords:** Prostate cancer, Active surveillance, Psychological distress, Online intervention, Web-based intervention, Digital intervention, Self-management, Anxiety, Person-based approach

## Abstract

**Objectives:**

To outline the planning, development and optimisation of a psycho-educational behavioural intervention for patients on active surveillance for prostate cancer. The intervention aimed to support men manage active surveillance-related psychological distress.

**Methods:**

The person-based approach (PBA) was used as the overarching guiding methodological framework for intervention development. Evidence-based methods were incorporated to improve robustness. The process commenced with data gathering activities comprising the following four components:

• A systematic review and meta-analysis of depression and anxiety in prostate cancer

• A cross-sectional survey on depression and anxiety in active surveillance

• A review of existing interventions in the field

• A qualitative study with the target audience

The purpose of this paper is to bring these components together and describe how they facilitated the establishment of key guiding principles and a logic model, which underpinned the first draft of the intervention.

**Results:**

The prototype intervention, named PROACTIVE, consists of six Internet-based sessions run concurrently with three group support sessions. The sessions cover the following topics: lifestyle (diet and exercise), relaxation and resilience techniques, talking to friends and family, thoughts and feelings, daily life (money and work) and information about prostate cancer and active surveillance. The resulting intervention has been trialled in a feasibility study, the results of which are published elsewhere.

**Conclusions:**

The planning and development process is key to successful delivery of an appropriate, accessible and acceptable intervention. The PBA strengthened the intervention by drawing on target-user experiences to maximise acceptability and user engagement. This meticulous description in a clinical setting using this rigorous but flexible method is a useful demonstration for others developing similar interventions.

**Trial registration and Ethical Approval:**

ISRCTN registered: ISRCTN38893965. NRES Committee South Central – Oxford A. REC reference: 11/SC/0355

## Key messages regarding feasibility

- At the start of this process, there were uncertainties around the feasibility of:
  - Reaching and recruiting the target group (men on active surveillance for prostate cancer)
  - Creating an accessible and acceptable intervention
- Key feasibility findings:
  - Men on active surveillance for prostate cancer would like additional support and are willing to take part in online and group-based sessions
- The research activities carried out in the intervention planning and development phases have optimised the acceptability of the proposed intervention.

## Background

Prostate cancer prevalence is high in the UK [1] and affects around one in eight men [2]. Treatment options include surgery, radiotherapy and hormone therapy, but for men with localised, low-risk prostate cancer, active surveillance (AS), a pathway that involves monitoring biological markers of the disease for progression, is also an option. AS aims to reduce overtreatment and comes without the unwanted side effects of interventional treatment, such as urinary incontinence and erectile dysfunction [3]. However, research has shown AS may have a negative impact on psychological wellbeing with patients experiencing heightened levels of anxiety [4–6], illness uncertainty, hopelessness [7] and distress [8].

Few studies have explored the unmet psychological needs of men on AS and ways in which wellbeing could be improved [9], and to our knowledge, there are no existing interventions available to support men on AS. The limited qualitative evidence suggests men on AS find AS-related information inadequate and inconsistent [9] and experience unmet psychological and emotional needs [9], and spousal support is important for AS acceptance [10]. Furthermore, anxiety and uncertainty are two key reasons men choose to discontinue AS and pursue interventional treatments in the absence of changes to tumour status [11, 12], risking treatment side effects. Research in the area of AS-related psychological wellbeing is vital to ensure patients have the information and tools that will allow them to better cope with this treatment pathway.

Best practice guidance recommends taking a ‘person-based’ approach (PBA) when developing behavioural interventions [13]. The PBA is an established method used to optimise interventions, providing a clear process to ground interventions in the perspectives and psychosocial context of the target user group. The PBA recommends comprehensive qualitative research with the target user group to explore their experiences and create a picture of the challenges they face, and in turn the things that are likely to influence a target behaviour. Exploration of the key beliefs target users hold, for example, about their condition, its management or treatment, is important to gain an understanding of what might facilitate or prevent change. Taking this approach, the target users’ needs and preferences can influence the content, structure and overall design of the intervention, ultimately improving the intervention’s feasibility, acceptability and user engagement [14]. Intervention developers are utilising PBA techniques increasingly [15–17]; however, there is a lack of literature providing replicable, worked examples of intervention development using the PBA.

This paper describes four previously published research activities, bringing them together to demonstrate how they contributed to the planning and development process of PROACTIVE, a psycho-educational intervention for men with localised prostate cancer on active surveillance, consisting of parallel group-support and web-based sessions. It aims to describe the methodology needed to plan such an intervention, presenting a demonstration of how to develop an intervention using a PBA. The resulting intervention has been trialled in a feasibility study, the results of which are published elsewhere [18].

## Methods

### Using the person-based approach to guide PROACTIVE planning

The PBA is flexible and non-prescriptive and can be utilised alongside evidence-based approach methods and theory. The PBA provides an established methodological framework for the intervention planning process [13].The approach consists of two key stages. The first involves gathering information from the target audience to gain an insight into what is wanted and needed, giving the researchers a deeper appreciation of the psychosocial context of the audience. This is an iterative process used to continually refine the intervention to promote acceptability and to encourage adherence and engagement.

The second stage is the creation of ‘key guiding principles’. These consist of (a) key intervention design objectives and (b) key distinctive features of the intervention needed to achieve objectives. These principles aim to keep the design and development process on track by providing the research team with a summary of objectives to be referred to at each stage of the process.

The planning process commenced with a systematic review and meta-analysis of depression and anxiety in PCa. This was an evidence gathering activity aiming to improve understanding about the prevalence and magnitude of psychological distress in men with PCa (not specific to AS).

Using the PBA framework, the following activities were subsequently conducted to facilitate our understanding of psychological distress specific to those on the AS pathway, the interventions that have been trialled previously and the supportive care needs of the target audience:

- A cross-sectional survey on depression and anxiety in active surveillance
- A review of existing interventions in the field
- A qualitative study with the target audience

These components facilitated the creation of key guiding principles and a logic model to guide the intervention development.

Table 1 shows the stages recommended by the PBA for intervention planning and development (columns 1 and 2) alongside an overview of the development of PROACTIVE (column 3) to show how the PBA process was implemented. Each activity will be described in depth in the next section.

**Table 1:** PBA implementation in PROACTIVE

Stage of intervention development and evaluation	Specific PBA activities useful at each stage	PROACTIVE planning, development and optimisation	Additional activities
1. Intervention planning	• Synthesise previous qualitative studies of user experiences of similar interventions • Carry out qualitative research to elicit user views of the planned behaviour changes and intervention (including relevant previous experience, barriers and facilitators)	• Review of existing interventions in the field • Qualitative study	• A systematic review and meta-analysis of depression and anxiety in prostate cancer (evidence-based approach activity)
2. Intervention design	• Use themes arising from the intervention planning stage to identify key issues, needs and challenges the intervention must address • Create guiding principles, comprising (a) key intervention design objectives and (b) key distinctive features of the intervention needed to achieve objectives	• Use themes from qualitative study to identify key issues, needs and challenges Proactive must address • Create guiding principles	
3. Intervention optimisation and evaluation of acceptability and feasibility	• Elicit and observe user reactions to every intervention element (e.g. using think­aloud techniques), iteratively modifying intervention to optimise acceptability and feasibility • Carry out detailed longitudinal mixed methods case studies of independent intervention usage	• Create Proactive prototype, conduct think aloud interviews and modify the intervention accordingly	

## Intervention planning

In this section of the paper, we describe the data gathering activities we undertook to consolidate our understanding of the issue (reduced psychological wellbeing in men on AS for PCa), and accumulate ideas about what might be helpful to this population. Table 2 provides an overview of these activities.

**Table 2:** Data gathering activities and relevance to PROACTIVE planning

Activity	Aim	Methods	Results	Relevance to PROACTIVE planning
**Review of the literature:** Systematic review and meta-analysis of depression and anxiety in prostate cancer (Full study published elsewhere [4])	An evidence-based approach activity to systematically review literature around depression and anxiety prevalence in patients with prostate cancer	Due to a lack of previous research about depression and anxiety in men specifically on the AS pathway, this review included men on other PCa treatment pathways. After de-duplication, 1130 articles were screened for eligibility, and 27 full journal articles were included giving a total sample size of 4494 prostate cancer patients	Anxiety and depression were highly prevalent, and levels varied throughout the course of the illness and according to treatment status. A pattern of depression and anxiety was identified showing rates were highest after diagnosis before treatment (depression: 17.27% (95% CI 15.06 to 19.72%), anxiety: 27.04% (95% CI 24.26 to 30.01%)), lowered during treatment (depression: 14.70% (95% CI 11.92 to 17.99%), anxiety: 15.09% (95% CI 12.15 to 18.60%)), and then raised again when treatment was complete (depression: 18.44% (95% CI 15.18 to 22.22%), anxiety: 18.49% (95% CI 13.81 to 24.31%))	Of the 27 articles included, only 4 involved AS patients [19–22]. The upper depression and anxiety prevalence rates reported within these articles were high, 17% and 21% respectively, indicating the need for further research into the psychological impact of AS, and an investigation into the use of a support tool
**Cross-sectional survey study:** Cross-sectional assessment of depression and anxiety prevalence in prostate cancer patients undergoing active surveillance (Full study published elsewhere [5])	To further explore the issue of heightened anxiety and depression in men with PCa, and provide a broader picture specific to AS	313 men being managed by AS for PCa across 7 UK urology centres were recruited. The primary outcome was the Hospital Anxiety and Depression Scale (HADS) [23]. The survey collected demographic data (age, employment, relationship, ethnic and educational status), to allow for cross-tabulation with anxiety and depression scores. Ethical approval was granted by the Berkshire Research Ethics Committee, reference 11/SC/0071	Results from this survey indicated a clinical depression prevalence of 12.5%, and clinical anxiety prevalence of 23% measured by the HADS. The results show a more than doubled depression prevalence, and almost tripled anxiety prevalence in men on AS for prostate cancer compared to men of a similar age in the general population (6% and 8% respectively [24]). Divorce was the only demographic predictor of higher anxiety and depression [5], indicating the family environment may need further investigation also	With the combined results from the systematic review and cross-sectional assessment indicating elevated levels of anxiety and depression, the research team concluded the levels of distress in men on AS for PCa needs to be addressed
**Review of existing interventions in the field:** A narrative literature review of supportive psychological interventions within prostate cancer (Full study published elsewhere [8])	To gain an insight into whether informational, psychological, emotional, or cognitive interventions can positively impact men with prostate cancer	Ideally this review would have focussed on AS patients, however, due to a paucity of interventions in this area, interventions for PCa not exclusive to AS were included. The interventions were categorised by delivery mode: delivered in a group-based environment, delivered over the phone, delivered over the internet, or delivered by other means. Intervention components and intervention effectiveness were considered in tandem (listed in Table 3) to enhance understanding of the components that may be effective in a new intervention	See Table 3 for a summary of the self-care interventions	See Table 3 for the implications for PROACTIVE
**Qualitative study with a sample of the target audience:** “They say most men die with and not from prostate cancer, but how do you live with it?” A qualitative interview study of the supportive care needs of patients on active surveillance (Full study published elsewhere [8])	The research team carried out a qualitative study with the aim of gaining a more in-depth and specific understanding of the supportive care requirements of prostate cancer patients being managed with AS	20 men on active surveillance were recruited from the prostate cancer clinic at Southampton General Hospital. Semi-structured qualitative interviews were conducted and analysed inductively using thematic analysis. Ethical approval was obtained from Oxfordshire Research Ethics Committee, reference 11/SC/0355)	Table 4 shows the key findings that emerged from the data. In summary, the men reported high levels of emotional distress, a lack of knowledge about their condition or how to self-manage it, and a desire for more information and support [8]	The results from this qualitative study indicate that men being managed with active surveillance would welcome specific additional psycho-educational support to help them better cope and manage with the burden of living with untreated prostate cancer. According to the data, a mixture of web-based support and group session support would be most appropriate

**Table 3:** Self-care psychological interventions targeting prostate cancer patients

Author(s)	Mode of delivery	Intervention description	Intervention components	Results/effectiveness/points of interest	Implications for PROACTIVE
Parker et al. (2009) [25]	Group-based environment	Pre-surgical stress management programme for men undergoing radical prostatectomy	2 × 90-min sessions that involved: • Learning relaxation skills • Guided imaginary rehearsals of surgery day • Discussion about fears and implementation of coping strategies 2 × booster sessions: on the morning of surgery; and 48 h post-surgery	Significantly less mood disturbance, cancer-related worries and physical side effects than controls. Effects maintained at 12 months 221 potential participants approached, 159 took part	Level of uptake suggests PCa patients are willing to take part in self-care interventions to improve psychological wellbeing
Penedo et al. (2004 and 2006) [26, 27]	Group-based environment	Cognitive behavioural stress management intervention with men who had received either radical prostatectomy or radiotherapy for PCa	10 week intervention with 2 h weekly sessions involving: • Implementing relaxation techniques • Utilising techniques such as identifying distorted thoughts • Goal setting • Utilising social support	Significant improvements in both general and PCa specific quality of life compared to controls	In PCa patients relaxation interventions are both well received and clinically effective
Carlson et al. (2003 and 2007) [28, 29]	Group-based environment	Standardised Mindfulness Based Stress Reduction (MBSR) course	8 weekly sessions incorporating: • Relaxation • Meditation • Gently yoga • Daily home practice (Note: Recruits not exclusively PCa)	Significant improvements in sleep, stress, anxiety, mood and fatigue	Results were not stratified by disease type, so hard to draw any strong implications for PROACTIVE; however, the study findings do support the notion of group-based support for cancer patients
Templeton and Coates (2004) [30]	Group based environment	Educational intervention	Brief, group-based, nurse-led single session for men being treated with hormone therapy for PCa. Participants were provided with an information booklet	Compared to controls, significant improvements in general and PCa-specific quality of life, PCa knowledge and satisfaction with care	Intervention effectiveness may not be dose related, and short interventions may be as effective as more time consuming programmes Information provision is valued by PCa patients The participants in this trial were receiving hormone treatment and those on AS may feel differently
Berglund et al. (2007) [31]	Group based environment	Psychosocial rehabilitation	7-week group based intervention 3 arms; information arm, physical activity arm, combined arm, plus a control group • Information arm led by PCa nurse and participants received information about what PCa is, treatment options, side effects and methods of dealing with urinary and erectile dysfunction • Physical activity arm led by physiotherapist and focussed on increasing daily exercise • Combined arm received both	No significant improvements in anxiety, depression or quality of life	Unclear why the intervention was unsuccessful, perhaps information needs to be combined with relaxation/stress management techniques
Lepore et al. (2003) [32]	Group based environment	Educational intervention	3 groups, intervention, intervention plus discussion, control group Intervention involved 6 1-h weekly group sessions involving: • Prostate cancer biology information • Treatment options • Managing side effects • Diet and nutrition • Stress, coping and relaxation	Both intervention arms showed significant improvements in PCa knowledge and experienced less sexual dysfunction compared to the control group. No significant improvements in depression	Depression baseline taken after treatment, low levels to start with so hard to draw conclusions for PROACTIVE from this
Bailey et al. (2004) [33]	Telephone	Intervention to manage uncertainty	This was designed for men on watchful waiting. 5 brief telephone consultations with a male PCa nurse, with week-long intervals. Telephone consultations around: • Re-framing negative thoughts • Managing uncertainty • Accepting watchful waiting	Significant improvements in quality of life and uncertainty management compare to controls	This study limited by high homogeneity in the sample, and watchful waiting patients may be different to AS patients
Chambers et al. (2013) [34]	Telephone	Psycho-educational intervention	5 brief telephone consultations with PCa patients around: • Cognitive reframing • PCa education • Management of side effects • Management of stress • Developing problem solving skills	Significant improvements in mental health and cancer related distress in younger patients with higher levels of education and income, but not in the rest of the sample	Heterogeneous sample compared to Bailey et al. (2004), and large sample recruited from multiple centres. Indicates telephone support may not be effective for some groups of PCa patients
Kazer et al. (2011) [35]	Internet	Intervention to manage uncertainty	5-week online intervention named “Alive and Well” for men on AS. Components included: • Cognitive reframing of negative thoughts • PCa and AS information • Lifestyle advice • Tailored emails	Significant improvement in 8 of the 12 quality of life subscales measured at the end of the intervention	An online-only intervention improve quality of life in men on AS
Osei et al. (2013) [36]	Internet	Intervention to improve quality of life	6-week online intervention for men radically treated for PCa involving: • Online support forum • PCa information • Support managing side effects	Significant improvements in quality of life, but not maintained at follow-up	Support may need to be more long term to improve outcomes beyond the study timeframe
Weber et al. (2004) [37]	Other—dyadic support	Social support intervention	Post-radical prostatectomy patients paired with men who had the same surgery 5 years previously. Men in the intervention arm met with long term survivors once a week for 8 weeks	Men in intervention arm reported significantly lower levels of depression and significantly higher levels of self-efficacy compared to controls. High attrition rate – all 8 sessions attended by every intervention arm participant	Dyadic support well adhered to Support from men who have had similar experiences can be effective in improving psychological outcomes

**Table 4:** Key findings from the qualitative study

Theme	Description	Quote
Emotional distress	All of the men interviewed showed increased levels of emotional distress to varying degrees due to being on active surveillance	“You are told that you have cancer but that it’s nothing to worry about and that all they are going to do is watch it to see how it grows. That just doesn’t make sense; if something can be done prior to it spreading, why isn’t it being done? It freaks you out.” (Interviewee 5, aged 72)
Lack of information and knowledge	Part of the reason for the increased levels of emotional distress seemed to be attributed to a lack of understanding and lack of information received about active surveillance	“If they had said what active surveillance is then perhaps I might have understood it a bit better, but they just said we will keep an eye on it every 6 months. You know, keeping an eye on it could mean a blood test, a meeting with the consultant, another biopsy. I was in the dark and it is the not knowing, the lack of information, that is what worries you”. (Interviewee 13, aged 71)
The need for additional support in the form of a support group	The men interviewed explained their access to Prostate Cancer experts is limited and therefore access to a group of men also under active surveillance would be helpful	“You know, you see the hospital doctors very infrequently so for me I always viewed my GP, who I have known for years, as my first port of call when I needed to better understand things relating to active surveillance and my tests and stuff. But the problem I found was, and I don’t mean to be rude, he [the GP] didn’t know any more about active surveillance than me so you are kind of left in this horrible place where no one has the ability to answer your questions or fears so meeting up with other chaps on active surveillance in a confidential and educationally focused group would be a real coup for me” (Interviewee 12, aged 58)
The need for additional support in the form of a website	Men wanted to play an active part in helping themselves and would value self-management information in the form of a website	“If prostate cancer specific information was available on the web, then that reassures people. Things like frequently asked questions, situations and symptoms to look out for, advice on what you can do to help manage it [prostate cancer], advice about diet, changes that I ought to be making. Things of that nature really, things to allow me to self-manage this [prostate cancer]” (Interviewee 1, aged 71)

## Intervention design

In this section of the paper, we describe how the data gathered in the ‘Intervention planning’ section was used to design the intervention, and how the creation of the ‘key guiding principles’ and ‘logic model’ facilitated this process.

### Key guiding principles

Using the information gathered during the intervention planning phase, the research team developed a set of key guiding principles, in line with the PBA approach. The purpose of this is to summarise the design objectives and how these will be achieved, to facilitate quick and easy reference throughout the planning and development phase to guide and focus the decision making around intervention content and design [13]. Table 5 outlines the three intervention design objectives along with the key features of the intervention designed to address each objective.

**Table 5:** Key guiding principles

Intervention design objective	Key features designed to address objective	Rationale for design objective and key features
1. To support men to manage the anxiety they experience due to being on active surveillance	The group sessions should provide support with anxiety by providing reassurance and normative information about others’ experiences, and encouragement in the development of coping skills (for example, social support mobilisation). The web component should complement the group sessions, enabling men to learn self-management strategies to take control of their anxiety. The web component should also signpost men to further reliable information available online	• All research conducted in the planning phase indicated emotional distress • The qualitative study with the target audience, and various previous interventions [25–30, 32] indicated group support may be beneficial • The qualitative study indicated a lack of reliable PCa and AS related information
2. To encourage and support a healthy lifestyle	The web component should provide information about diet, physical activity and stress management to encourage a healthy lifestyle. Asking the men to set weekly goals could encourage them to utilise the information and implement changes to benefit their health. Relaxation techniques to be demonstrated in the group sessions, and the lifestyle information reinforced	• Target audience in qualitative study requested self-management tools/advice
3. To maximise engagement in the programme	In order to encourage participation from men who might usually feel uncomfortable participating, and to minimise withdrawal from the group sessions, the group sessions need to be facilitated in a way that encourages active participation, mutual understanding, respect and help-seeking behaviour (for example, demonstrating an interest for further information). To encourage men who might otherwise not participate the group facilitator needs to sensitively encourage patients to identify their emotional state and feelings	• Important to maximise engagement in the programme to maximise change/improvements

### Developing a logic model

**Fig. 1:**
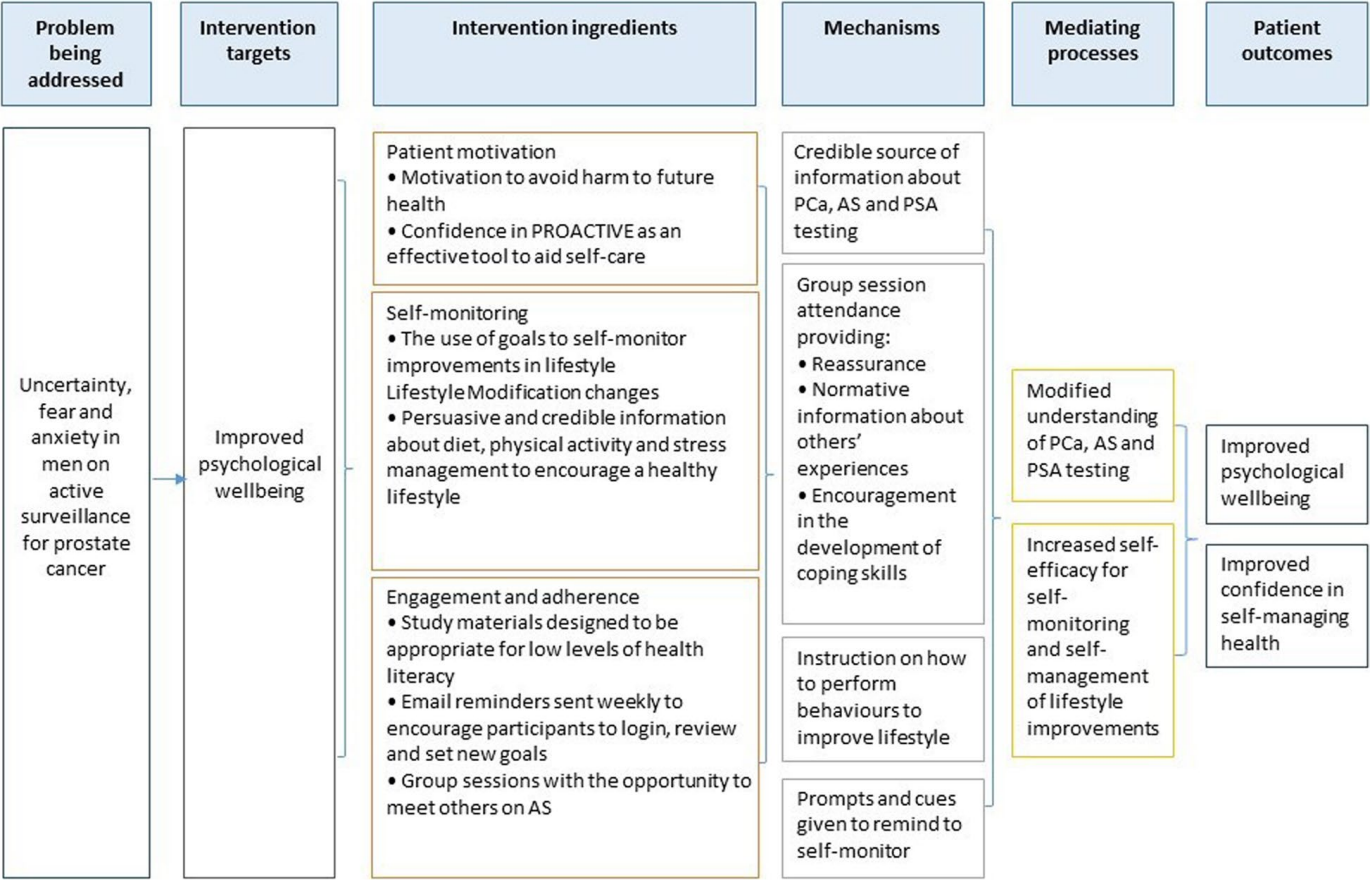
Logic model

The MRC complex intervention guidelines [38] recommend the development of a logic model to outline the hypothesised causal mechanisms involved in bringing about change in men on AS for PCa. The logic model (Fig. 1) demonstrates how we anticipate the intervention will result in improved psychological wellbeing.

### Public and patient involvement.

Three patient and public involvement (PPI) contributors with PCa were involved in the intervention planning and development process. The research team met with the contributors every 4–8 weeks to provide progress updates and gain feedback on ideas and written materials. PPI contributors reviewed and commented on the online and workshop content and were involved in all key decisions. The involvement of the PPI contributors ensured developing study materials were likely to be acceptable, understandable and relevant to the target audience.

## Results

### The PROACTIVE prototype

The prototype intervention was named PROACTIVE – ‘PROstate ACTIVE surveillance support’. The intervention consisted of two parts:

1. An online programme consisting of six sessions designed to be completed on a weekly basis.
2. A face-to-face group support programme with three sessions, spread across six weeks, held fortnightly and each lasting 60–90 min.

**Fig. 2:**
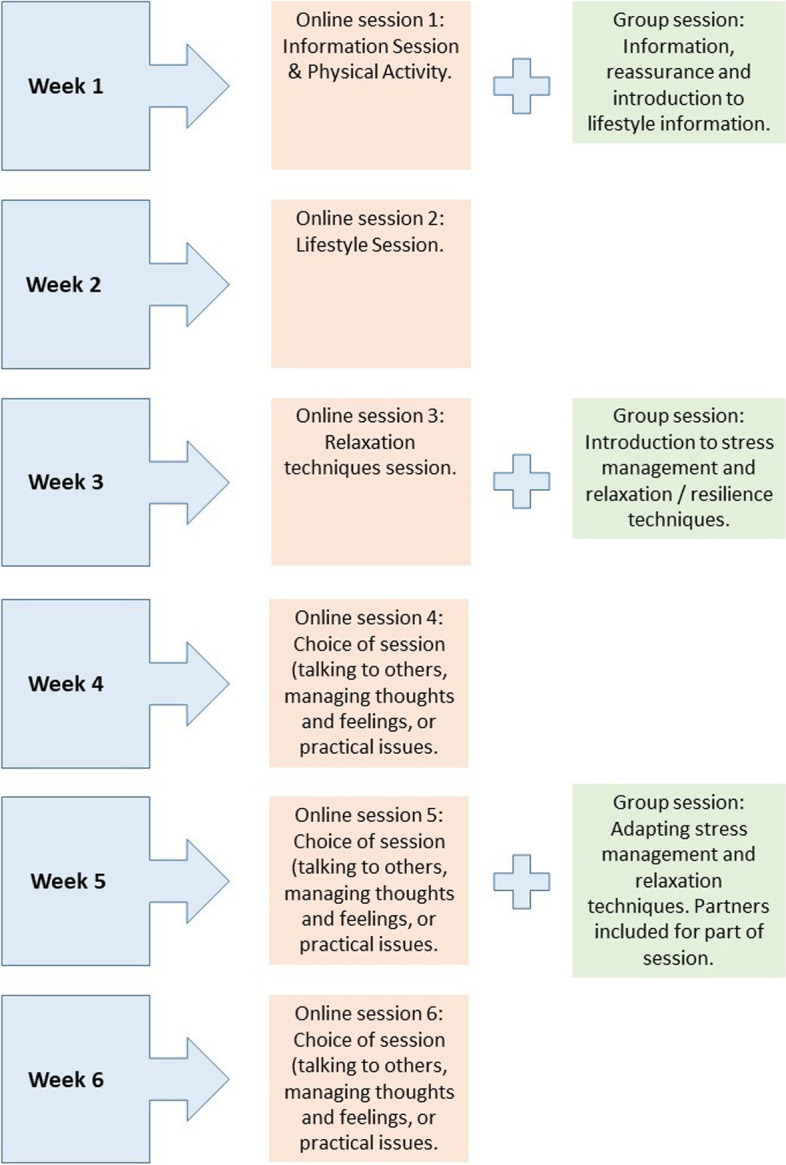
The PROACTIVE intervention

The web-based programme and the group support sessions interlink and are designed to run in parallel over 6 weeks, complementing each other; for example, the online sessions introduce topics that will be further discussed in the following group session, and the online sessions reinforce the information covered in previous group sessions. Figure 2 shows the intervention as a whole over the 6 week time period. See Table 6 for a detailed description of the session content.

### The intervention

Table 6 details the content of each of the web-based and face-to-face group sessions delivered over the 6-week period.

**Table 6:** The intervention

Week	Delivery mode	Session title	Content	Why was this included?	Target behaviour(s)
1	Web-based	Introduction and physical activity	Introduction to the website Information about active surveillance, Prostate Specific Antigen (PSA) testing, prostate cancer in general and prostate cancer statistics with printable information sheets Section on physical activity providing information about the benefits of increasing physical activity and ways to go about it	Target audience in qualitative study requested: • Reliable information • Self-management tools/advice	• Engagement with the programme • Increased PCa knowledge • Motivation to increase physical activity
1	Face-to-face group session	Information, reassurance and introduction to lifestyle information	Provision of information to ensure patients are fully informed about their condition and treatment plan. Time for patients to ask questions included Provision of reassurance and comfort by identifying areas of concern related to active surveillance, targeting possible frustration related to inadequate information Introduction to lifestyle (diet and exercise) information	Target audience in qualitative study requested: • Reliable information • Self-management tools/advice • Social support from other men on the AS pathway	• Engagement with the programme • Increased PCa knowledge • Motivation to improve lifestyle
2	Web-based	Foods to eat	Advice about foods to eat. General healthy eating advice, with some specific prostate cancer diet information	Target audience in qualitative study requested self-management tools/advice	• Improved nutrition knowledge Motivation to improve diet
3	Web-based	Relaxation & Resilience techniques	Two relaxation techniques described with step-by-step instructions	Target audience reported: • Emotional distress • Desire for self-management tools/advice Relaxation techniques aim to provide men with self-management tools to address high levels of self-reported emotional distress	• Improved confidence in the self-management of emotional distress • Improved self-management of emotional distress
3	Face-to-face group session	Introduction to stress management and relaxation / resilience techniques	• Identifying forms of coping • Presentation of 2 relaxation and resilience techniques Discussion of issues and concerns related to lifestyle and active surveillance related information	Target audience reported: • Emotional distress • Desire for self-management tools/advice • Social support from other men on the AS pathway	• Improved confidence in the self-management of emotional distress • Improved self-management of emotional distress • Improved confidence in improving lifestyle
4	Web-based	Talking to family/friends/professionals^a^	Advice and ideas about how to approach the subject of PCa and/or AS with friends and family How to get the most out of specialist consultations	Target audience reported: • Emotional distress • Lack of reliable information Improved PCa/AS related communication with friends and family may reduce emotional distress Improved communication in specialist consultations may help fill any gaps in knowledge or understanding	• Improved PCa related communication with family and friends • Improved communication in specialist consultations to ensure patient questions are answered adequately
5	Web-based	Thoughts and feelings^a^	Addresses distressing thoughts and feelings the men may be experiencing. Provides advice about how to manage and/or reduce these thoughts and feelings	Target audience reported: • Emotional distress • Desire for self-management tools/advice	• Improved confidence in the self-management of emotional distress • Improved self-management of emotional distress
5	Face-to-face group session Partners invited to the first 20 min of this session	Adapting stress management and relaxation techniques	• Opportunity for the partners of the participants to ask questions and share experiences. Partners to leave the session after 20 min • Reinforcing and adapting relaxation and resilience techniques for future use • Discussion/feedback about the PROACTIVE programme • Opportunity for participants to gain clarity about anything covered in the programme	Partners included to: • Promote PCa/AS related communication • To ensure gaps in knowledge and understanding are filled for both the men and their partners Reinforcement of all that has been learnt over the programme to provide confidence in moving forward with self-management tools Provision of social support from other men on the AS pathway	• Confidence in implementing self-management tools moving forward • Confidence in PCA and AS knowledge and understanding
6	Web-based	Daily life; money and work^a^	Covers advice about practical issues (for example, paying for hospital parking) and provides information about dealing with work after receiving a diagnosis	Practical advice to aid self-management	• Improved confidence in managing diagnosis in work
1–6		Goal and plan setting / Goal and plan reviewing	1–3 weekly goals, with a plan to go with each one. Goals and plans reviewed on a weekly basis	Goal setting provides a way of self-monitoring progress and provides some accountability	
1–6		Web links	Website links to useful resources related to each session	Target audience reported a lack of reliable information. Links provide further information from credible sources	

^a^These three online sessions could be completed in any order

### Think aloud interviews to refine PROACTIVE

Identified by the PCaSO charity (Prostate Cancer Support Organisation), 2 men with prostate cancer took part in think aloud interviews. This process involved each participant working their way through the PROACTIVE prototype whilst simultaneously speaking aloud their thoughts about the programme. Statements such as ‘can you tell me what you think about this page?’ and ‘can you tell me why you chose that option?’ were used as prompts to elicit participants’ opinions on the intervention. Interviews were audio recorded, and participants’ thoughts and opinions were collated and used to amend the prototype to be used in the feasibility study. Table 7 provides a summary of these changes.

**Table 7:** Summary of changes from think aloud interviews

Comments from think aloud	Action
Various aesthetic suggestions were made, for example, removing/adding pictures, less information on each page, centralising headings	All suggestions were discussed within the research team and the majority were implemented
Suggestion to add in a quick summary of all sessions as part of the introduction	Added as suggested
Minor wording changes to information sheets and sessions suggested	Implemented as suggested
Physical activity section needs to be relevant to PCa	Physical activity advice was included, and recommendations in line with NHS guidance. The research team added information to explain how improving physical activity can improve wellbeing and general health
Physical activity session assumes all participants are unfit, amend wording to remove this assumption	Implemented as suggested
Add more examples of goals	Added as suggested
Add in the benefits of talking to others	Added as suggested
Section about talking to professionals implies there will be problems	Wording adjusted to remove implication
Add statement that doctors / nurses will not be embarrassed by certain topics	Added as suggested
Make goal page printable	Actioned as suggested

### PROACTIVE ready for feasibility study

**Fig. 3:**
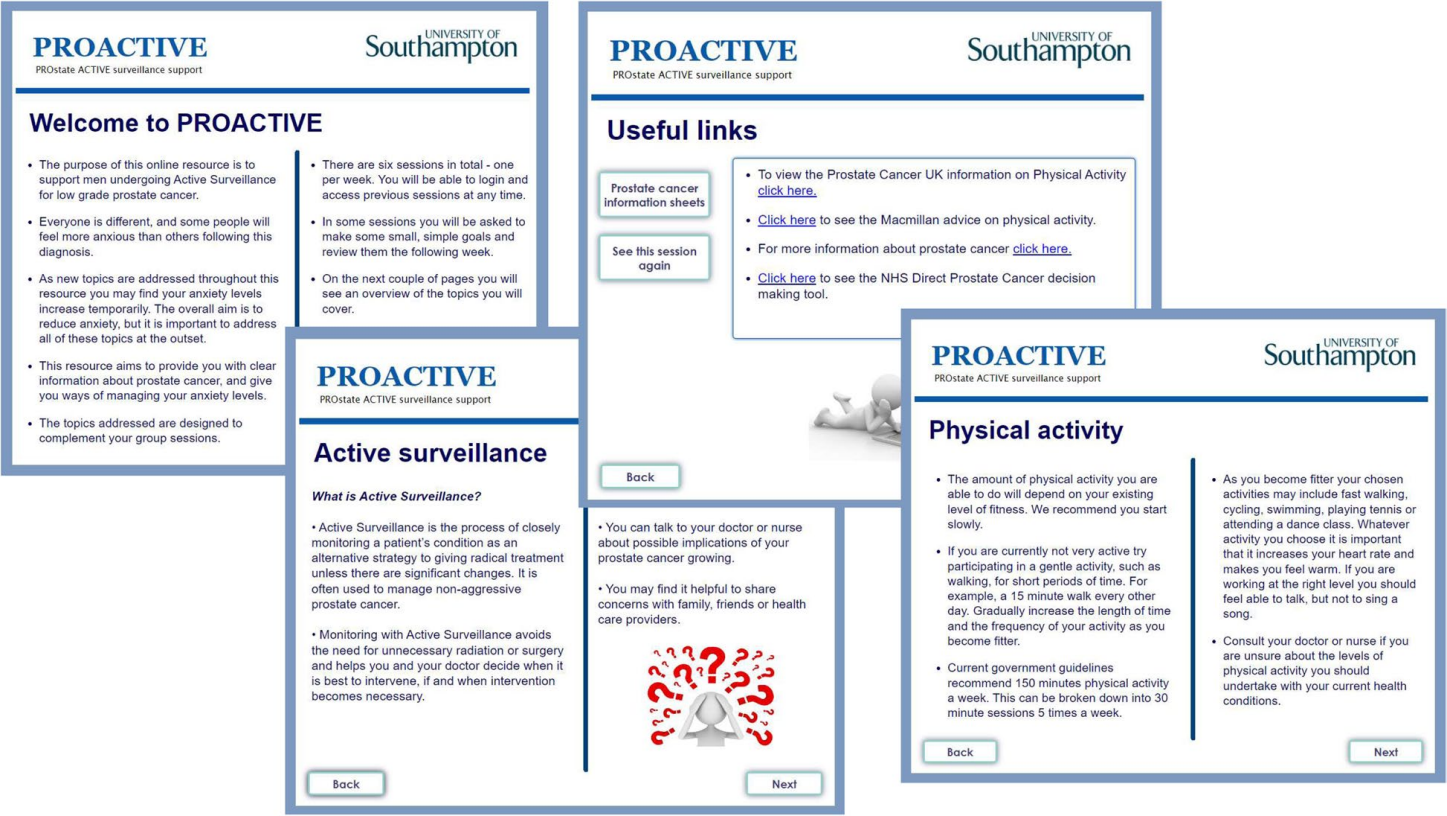
PROACTIVE screenshots

The amended intervention became the final version for the feasibility study. Figure 3 shows some screenshots from the web-based sessions.

The feasibility study has been conducted, and the results published elsewhere [18].

## Discussion

### Summary

This paper demonstrates how we used the PBA to develop an Internet- and group-based psycho-educational behavioural intervention for men on AS for PCa. The process of gathering, understanding and utilising target-user needs and perspectives has long been viewed as essential within the eHealth research community [39–41]. Intervention developers in the area of eHealth have undertaken this task in a variety of ways [42, 43]; however, until the publication of the PBA, there was no standardised approach or process to follow. Guided by the PBA, the researchers gained a context-specific understanding of the intervention elements likely to be needed to maximise participant acceptability and engagement.

Each component of our approach (see Table 1) added a valuable contribution to the development process. The systematic review and meta-analysis of depression and anxiety in prostate cancer provided a broad understanding of the prevalence of these conditions in PCa patients, and confirmed the dearth of literature specific to AS. Beginning to fill this gap in the literature, the cross-sectional survey on depression and anxiety in AS allowed us to narrow down the treatment pathway diversity in previous studies and focus on men on AS. Men in this survey displayed significant levels of distress, reinforcing the value of the proposed intervention. Reviewing existing interventions in the field provided a way of seeing what has and has not been successful in the past, and an understanding of the elements that may increase the success of the proposed intervention. The qualitative study gave us the opportunity to explore the supportive care needs of this user group in an in-depth way providing focussed direction for the intervention and a way of identifying any gaps.

Integrating the results from these 4 components we were able to create a set of key guiding principles. The key guiding principles summarised the design objectives and provided focus for decision making. Our logic model provided a visual representation of how the intervention might work, displaying the intervention ingredients and how these translate into the causal mechanisms likely to bring about change.

Armed with the understanding gained from the abovementioned processes, we were able to create the prototype PROACTIVE intervention. The think-aloud interviews provided further clarity, and the minor changes made due to the results of these interviews improved our confidence in the intervention.

This methodological approach is rigorous but flexible. For other interventions, the stages and processes may differ depending on the context of the intervention [15, 16, 44, 45], for example, if there is already a large base of existing qualitative research, new qualitative research may not be necessary.

### Strengths, limitations and future research

Treatment for PCa is a rapidly changing and advancing field, for example, medical technology and the accuracy of diagnostic tests continually being improved. For this reason PCa interventions (including PROACTIVE) would need to be updated regularly to stay current and accurate. The advantage of this development process is that it has produced a core product based on a rigorous transparent process with clear guiding principles that can easily be shared, adapted and updated, negating the need to repeatedly start from scratch.

The PBA recommends incorporating behavioural science into the development of interventions by integrating a ‘theory-based’ approach with the PBA processes as best practice. In this instance, this was not possible due to time and resource constraints. Further research conducting theory-based processes, and mapping the findings to the intervention, identifying any gaps, would be beneficial to potentially strengthen the intervention.

## Conclusion

This paper outlines the stages we followed using the PBA to develop the PROACTIVE intervention. The planning and development process is key to successful delivery of an appropriate accessible intervention. This meticulous description in a clinical setting using this rigorous but flexible method is a useful demonstration for others developing similar interventions.

## Data Availability

The datasets supporting the conclusions of this article are included within the article and its additional files.
